# Whole heart atherosclerosis volume and risk for major adverse cardiovascular events across the clinical likelihood for obstructive CAD; the CONFIRM2 study

**DOI:** 10.1016/j.ajpc.2026.101712

**Published:** 2026-06-24

**Authors:** Alexander van Rosendael, Rine Nakanishi, Jeroen J. Bax, Gianluca Pontone, Saima Mushtaq, Ronny R. Buechel, Christoph Gräni, Gudrun Feuchtner, Pietro G. Lacaita, Amit R. Patel, Cristiane C. Singulane, Andrew D. Choi, Mouaz Al-Mallah, Daniele Andreini, Ronald P. Karlsberg, Geoffrey Cho, Carlos E. Rochitte, Mirvat Alasnag, Ashraf Hamdan, Filippo Cademartiri, Erica Maffei, Hugo Marques, Pedro M. Gonçalves Pereira, Himanshu Gupta, Martin Hadamitzky, Omar Khalique, Dinesh Kalra, James D. Mills, Nick S. Nurmohamed, Paul Knaapen, Matthew Budoff, Kashif Shaikh, Enrico Martin, David M. German, Maros Ferencik, Andrew C. Oehler, Roderick Deaño, Prashant Nagpal, Marly van Assen, Carlo Nicola De Cecco, Vasileios Kamperidis, Borek Foldyna, Jan Michael Brendel, Victor Y. Cheng, Kelley Branch, Marcio Bittencourt, Sabha Bhatti, Venkateshwar Polsani, George Wesbey, Rhanderson Cardoso, Ron Blankstein, Augustin Delago, Amit Pursnani, Amro Alsaid, Vasvi Singh, Melissa Aquino, Ibrahim Danad

**Affiliations:** a Department of Cardiology, University Medical Center Utrecht, Utrecht, the Netherlands; b Department of Cardiovascular Medicine, Toho University Graduate School of Medicine, Tokyo, Japan; c Department of Cardiology, Leiden University Medical Center, Leiden, the Netherlands; d Department of Perioperative Cardiology and Cardiovascular Imaging, Centro Cardiologico Monzino IRCCS, Milan, Italy; e Department of Biomedical, Surgical and Dental Sciences, University of Milan, Milan, Italy; f Perioperative Cardiology and Cardiovascular Imaging Department, Centro Cardiologico Monzino IRCCS, Milan, Italy; g Department of Nuclear Medicine, Cardiac Imaging, University Hospital and University of Zurich, Zurich, Switzerland; h Department of Cardiology, Inselspital, Bern University Hospital, University of Bern, Bern, Switzerland; i Department of Radiology, Medical University of Innsbruck, Innsbruck, Austria; j Division of Cardiovascular Medicine, University of Virginia, Charlottesville, VA, USA; k Department of Cardiology and Radiology, George Washington University, Washington, District of Columbia, USA; l Department of Cardiology, Houston Methodist, Houston, TX, USA; m Division of University Cardiology, IRCCS Galeazzi Sant'Ambrogio, Department of Biomedical and Clinical Sciences, University of Milan, Milan, Italy; n Cardiovascular Research Foundation of Southern California, Beverly Hills, CA, USA; o Smidt Heart Institute, Cedars-Sinai Medical Center, Los Angeles, CA, USA; p David Geffen School of Medicine, University of California, Los Angeles, CA, USA; q Heart Institute, InCor, University of Sao Paulo Medical School, Sao Paulo, Brazil; r Cardiac Center, King Fahad Armed Forces Hospital, Jeddah, Saudi Arabia; s Department of Cardiology, Rabin Medical Center, Petah Tikva & Tel-Aviv University, Tel-Aviv, Israel; t Istituto di Ricovero e Cura a Carattere Scientifico SYNLAB Servizi Diagnostici Nucleari, Naples, Italy; u Department of Radiology, Istituto di Ricerca e Cura a Carattere Scientifico SYNLAB SDN, Naples, Italy; v UNICA, Unit of Cardiovascular Imaging, Hospital da Luz, Imaging Department, Católica Medical School, Lisbon, Portugal; w Cardiac Imaging, Heart and Vascular Institute, Valley Health System, Paramus, NJ, USA; x Department of Cardiovascular Radiology and Nuclear Medicine, TUM University Hospital German Heart Center, Munich, Germany; y Division of Cardiovascular Imaging, St. Francis Hospital and Heart Center, Roslyn, NY, USA; z Division of Cardiology, Department of Medicine, University of Louisville School of Medicine, Louisville, KY, USA; aa Department of Medicine, Division of Cardiovascular Diseases & Hypertension, Robert Wood Johnson Medical School, Rutgers University, New Brunswick, NJ, USA; ab Department of Vascular Medicine, Amsterdam UMC, University of Amsterdam, Amsterdam, the Netherlands; ac Department of Cardiology, Amsterdam UMC, Vrije Universiteit Amsterdam, Amsterdam, the Netherlands; ad The Lundquist Institute, Torrance, CA, USA; ae University of Tennessee Medical Center, Knoxville, TN, USA; af Division of Cardiology, MercyOne-Iowa Heart Center, Des Moines, IA, USA; ag Knight Cardiovascular Institute, Oregon Health & Science University, Portland, OR, USA; ah Allegheny Health Network Cardiovascular Institute, Allegheny Health Network, Pittsburgh, PA, USA; ai Department of Medicine, Division of Cardiovascular Medicine, University of Wisconsin School of Medicine and Public Health, Madison, WI, USA; aj Department of Radiology, University of Wisconsin School of Medicine and Public Health, Madison, WI, USA; ak Department of Radiology and Imaging Sciences, Emory University, Atlanta, GA, USA; al 1st Cardiology Department, Medical School, Aristotle University of Thessaloniki, Thessaloniki, Greece; am Department of Radiology, Cardiovascular Imaging and Research Center, Massachusetts General Hospital and Harvard Medical School, Boston, MA, USA; an Minneapolis Heart Institute, Minneapolis, MN, USA; ao Division of Cardiology, University of Washington, Seattle, WA, USA; ap Heart and Vascular Institute, University of Pittsburgh Medical Center, Pittsburgh, PA, USA; aq National Institute of Cardiovascular Diseases, Karachi, Pakistan; ar Piedmont Heart Institute, Atlanta, GA, USA; as Department of Cardiovascular Computed Tomography, Scripps Clinic, La Jolla, CA, USA; at Division of Cardiovascular Medicine, Brigham and Women's Hospital, Boston, MA, USA; au Medical Data Research Collaborative, London, United Kingdom; av Department of Medicine, Mount Auburn Hospital, Harvard Medical School, Cambridge, MA, USA; aw Cardiology, Endeavor NorthShore Cardiovascular Institute, Evanston, IL, USA; ax University of Chicago, Pritzker School of Medicine, Chicago, IL, USA; ay Department of Cardiac Imaging, Baylor Scott and White, The Heart Hospital Plano, Plano, TX, USA; az Midwest Heart & Vascular Associates, Kansas City, MO, USA; ba Cleerly, Inc, Denver, CO, USA; bb Department of Cardiology, Radboud University Medical Center, Nijmegen, the Netherlands

**Keywords:** Coronary plaque, Risk stratification, AI

## Abstract

**Introduction:**

The totality of atherosclerosis from the coronary tree can be measured with quantitative coronary CT and is increasingly being recognized as important marker for future cardiovascular events. We evaluated the risk and absolute event rates of plaque volume staging systems and provided analyses according to the clinical likelihood of obstructive CAD.

**Methods:**

CONFIRM2 is an ongoing, global, multicenter, observational registry that included patients with clinically indicated coronary CT angiography and follow-up for major adverse cardiovascular events (MACE). Patients without cardiac symptoms and prior CAD were excluded. Atherosclerosis was quantified across all coronary segments by AI-QCT. Total plaque volume (TPV) was categorized into non-calcified plaque (NCPV, HU <350) and calcified (HU>350). The primary end point was the incidence rate of MACE over at 4 years and included all-cause mortality, myocardial infarction, stroke, congestive heart failure, late revascularizations (occurring >90 days post index CCTA), and hospitalization for unstable angina. Secondary endpoint included Death, MI, and stroke. The prognostic value of TPV and NCPV was evaluated across the pretest likelihood according to the ESC Risk Factor Weighted Likelihood (ESC RF-CL).

**Results:**

A total of 6061 patients (mean age 58.6 ± 12.1 years, 48.6% male) were included and the mean follow-up duration was: 4.4 ± 1.8 years. According to the ESC RF-CL for obstructive CAD, patients were classified as very-low, low, and moderate risk in 36.0%, 43.0%, and 22.0%, and obstructive CAD rates were 6.8%, 15.7%. and 28.2, respectively. Within the total cohort, TPV >0–250, 250–750, and >750 mm^3^ were associated with MACE: HR 2.5 (95% CI 1.1, 5.6), HR 10.1 (95% CI 4.4, 22.9), and HR 15.9 (95% CI 6.7, 38.0) compared with TPV=0. According to RF-CL very-low, low, and moderate, the MACE rates were: 0.7, 2.9, 0.0% for TPV=0 mm^3^; 2.3%, 3.6%, 4.6% for TPV >0–250 mm^3^; 10.3%, 12.3%, 13.5% for TPV 250–750 mm^3^; and 18.8%, 16.7%, and 19.8% for TPV>750 mm^3^. Findings were consistent for the secondary endpoint.

**Conclusion:**

Among a large cohort of symptomatic patients evaluated by coronary CT angiography, the burden of atherosclerosis by AI-QCT was the main driver for cardiovascular events. Absolute event rates were consistently higher by each plaque volume stage, and consistent across the clinical likelihood subgroups for obstructive CAD. While quantitative atherosclerosis was present in the majority of patients, risk increased significantly from 250 mm^3^ of TPV during intermediate term follow-up.

## 1 Introduction

Coronary CT angiography is increasingly being used as a first-line diagnostic test for patients with suspected coronary artery disease (CAD) [1]. Visualization of the coronary arteries enables assessment of atherosclerosis within the entire coronary tree, which is recognized as a strong determinant of future cardiovascular events. Previous large CT registries that utilized visual plaque interpretation have shown a graded increase in risk according to the extent of CAD, regardless of the presence or absence of obstructive stenosis [[2], [3], [4], [5]]. For instance, Bittencourt et al. demonstrated that extensive non-obstructive CAD detected by CCTA was associated with rates of cardiovascular death or myocardial infarction comparable to those observed in patients with obstructive disease [[5], [6], [7]]. Although the relative risk associated with obstructive disease is highest, most events occur in patients with non-obstructive stenosis who were scanned several years before [8]. This has prompted a growing focus on quantifying the overall atherosclerotic burden, which is now feasible using quantitative AI-guided software tools.

Previous studies applying quantitative CT to large prospective cohorts have demonstrated that the strongest prognostic value lies in the assessment of non-calcified or low-attenuation plaque. In the ICONIC case-control study of patients who developed acute coronary syndrome after baseline coronary CT, non-calcified plaque with the lowest density conferred the highest risk, while the highest density calcification was associated with reduced risk [9,10]. Similarly, quantitative plaque evaluation from the SCOT-HEART trial revealed that low-attenuation plaque was the most powerful risk marker when compared with calcium score and stenosis severity [11]. To date, studies have shown strong associations between total or non-calcified atheroma volume and clinical outcomes, but limited data exist on the plaque volume magnitudes corresponding to absolute event rates.

CONFIRM 2 is a large, global registry of patients undergoing clinically indicated coronary CT with quantitative assessment of atherosclerosis by Artificial Intelligence–based Quantitative CT (AI-QCT). The aim of the present study was to evaluate the ability of quantitative atherosclerosis assessment to stratify risk in a large population and to provide guidance on the absolute event rates associated with different plaque volume magnitudes. Specifically, we analyzed patients categorized as having very-low, low, or moderate likelihood of obstructive CAD, as defined by the ESC guidelines according to age, sex, risk factors, and symptom typicality.

## 2 Methodology

### 2.1 Study design and patient population

The CONFIRM 2 (COroNary CT Angiography Evaluation For Evaluation of Clinical Outcomes: An InteRnational, Multicenter Registry) is an ongoing multicenter, international, observational cohort study. Detailed descriptions of the study design have been published previously [12,13]. The primary aim of the CONFIRM2 registry is to assess the prognostic value of AI based quantitative measures of coronary atherosclerosis including plaque burden, morphology and composition as well as the degree of luminal obstruction for predicting MACE. Participating sites prospectively and/or retrospectively included sequential patients with a clinically-indicated coronary CT of ≥ 64-detector rows. Exclusion criteria for enrollment were absence of CT-data or follow-up information for clinical events, pregnancy, or a non-cardiac illness with life expectancy <2 years. For this analysis, we included patients with cardiac symptoms and without prior CAD (defined as a prior myocardial infarction, prior percutaneous coronary intervention or coronary artery bypass grafting or known ≥50% stenosis on invasive coronary angiography) and who had at least 3 years of follow-up for clinical events. This study was conducted in accordance with the Declaration of Helsinki. Institutional review board approval was obtained at each participating center. The design and rationale of the CONFIRM 2 registry have been described previously [12].

### 2.2 Clinical data collection

Cardiovascular risk factors were systematically collected from the electronic medical record baseline prior to CCTA. Hypertension was defined as a blood pressure of ≥140/90 mmHg, a documented history of hypertension, or current treatment with antihypertensive medications. Diabetes mellitus was defined by a prior physician diagnosis and/or the use of insulin or oral hypoglycemic agents. Dyslipidemia was defined as either untreated dyslipidemia or current treatment with lipid-lowering medications. Smoking history was classified as positive if the patient was a current smoker or had ceased smoking within three months of testing. Family history of CAD was based on patient self-report. Symptom presentation was categorized as typical chest pain, atypical chest pain, non-cardiac pain, dyspnea, palpitations, syncope, other, or asymptomatic. The Risk Factor Weighted Clinical Likelihood (RF-CL) pre-test likelihood for obstructive CAD was calculated using age, sex, symptoms, and the number of traditional risk factors, as defined in the ESC guidelines for the management of chronic coronary syndromes [1].

### 2.3 Image acquisition and analysis

CT-scans were performed using various CT platforms, all of which met the requirement of employing multislice CT scanners with ≥ 64 slices. The imaging protocol adhered to the guidelines set forth by the Society of Cardiovascular Computed Tomography [14]. Patient preparation, data acquisition, and image analysis were conducted according to the institutional policies of the participating sites.

Quantitative coronary atherosclerosis evaluation was performed for every coronary artery and its branches using an automated U.S. Food and Drug Administration–cleared AI-enabled software platform (Cleerly Labs, Cleerly Inc., Denver, CO, USA). The AI-software employs validated convolutional neural networks for image quality assessment, coronary segmentation and labeling, lumen wall evaluation, vessel contour determination, and plaque characterization. Prior validation of the software has been documented in multicenter trials comparing it to expert consensus, quantitative coronary angiography, fractional flow reserve, and intravascular ultrasound [[15], [16], [17]]. This analysis was performed on all CT-scans and only coronary segments with a diameter of ≥1.5 mm were included. Each segment was assessed for the presence of coronary atherosclerosis, defined as any tissue structure ≥1 mm^3^ within the coronary artery wall that could be distinguished from surrounding epicardial tissue, epicardial fat, or the vessel lumen. Plaque volumes (in mm³) were calculated for each coronary lesion and summed to determine the total plaque volume at the patient level. Plaque types are categorized based on Hounsfield unit (HU) ranges: noncalcified plaque (NCPV) as HU between 30 and 350, and calcified plaque (CP) as >350 HU. High risk plaque (HRP) was defined as a lesion with LAP ≥ 2 mm^3^ and a remodeling index of >1.1. This definition is based on Omori et al. [17], since this magnitude of LAP and vessel remodeling correlated most strongly with near-infrared spectroscopy-intravascular ultrasound detected lipid core. Plaque burden normalized to the vessel volume was also reported as percentage atheroma volume (PAV), calculated as (Plaque Volume/Vessel Volume) × 100%. The vessel volume includes all coronary segments with a diameter ≥1.5 mm, regardless of plaque presence, differing from other quantitative CT-software that may include only segments containing plaque. The quantification software detects small amount of plaque which results in a low prevalence of completely normal CCTA and high prevalence of non-obstructive stenosis. At the Core Lab, exclusions are applied at the segment level, not automatically at the vessel or exam level. If a given coronary segment is non-assessable due to motion artifacts, low contrast attenuation, beam hardening, or other image artifacts, only that segment is excluded, while the remainder of the vessel is analyzed if interpretable. Cases are excluded on a case by case basis, for instance when segmental exclusions significantly impact the length of the evaluable vessel or proximal segments. Finally, a credentialed and trained radiologic technologist provides quality assurance overview of the AI-analysis.

### 2.4 End points

The primary end point was the incidence rate of MACE at 4 years. MACE was defined as a composite of all-cause mortality, myocardial infarction, stroke, congestive heart failure, late revascularizations (occurring >90 days post index CCTA), and hospitalization for unstable angina. Definitions are provided in the rationale and design study [12]. Death status was confirmed through signed verification forms submitted by the local principal investigators. The secondary endpoint was a composite of all-cause mortality, stroke, and myocardial infarction. All endpoints were recorded and adjudicated by a Clinical Events Committee.

### 2.5 Statistical analysis

Descriptive data are presented as median and interquartile range (IQR) for non-parametric data and as mean ± standard deviation for parametric data. Categorical variables are expressed as absolute numbers and percentages. Cox proportional hazards regression was used for outcome analyses. Kaplan-Meier curves were plotted to visualize event-free survival. The following coronary CT derived metrics were evaluated for their prognostic value: percent diameter stenosis, number of >50% stenoses, total plaque volume (TPV), noncalcified plaque (NCPV) volume, and presence of a proximal left anterior descending (LAD) or left main (LM) stenosis >50%. A previously published CAD staging system based on TPV was applied as described by Min et al., using the following categories: 0, >0–250, >250–750, and >750 mm³ [18]. For NCPV, with categories defined as 0–20, >20–125, >125–375, and >375 mm³; NCPV volume was also analyzed by tertiles. NCPV <20 was combined with absence of plaque to avoid potential image noise as plaque. In addition, a > 30% stenosis combined with a TPV threshold of 250 mm³ was used to refine risk stratification by combining stenosis and plaque burden. Diameter stenosis was categorized as 0%, 1–24%, 25–49%, 50–69%, 70–99%, and total occlusion (100%), and RF-CL–based risk categories were defined as very low, low, and moderate. All statistical analyses were performed using SAS 9.4 (SAS Institute Inc., Cary, NC) A p-value < 0.05 was considered as statistically significant.

## 3 Results

### 3.1 Patients

The study flow chart is provided in Appendix Fig. 1. A total of 6061 patients (mean age 58.6 ± 12.1 years, 48.6% male) were included and the mean follow-up duration was: 4.4 ± 1.8 years. Patients were predominantly white (83.1%), had baseline diabetes in 25.1%, and presented mostly with atypical chest pain (25.9%), Table 1. According to the ESC risk factor weighted clinical likelihood (RF-CL) for obstructive CAD, patients were classified as very-low, low, and moderate risk in 36.0%, 43.0%, and 22.0%. With higher RF-CL, patients were significantly older (52.1, 60.6, and 65.1 years, p<.0001), more often male (23.2%, 50.4%, 86.5%, p<.0001), had a higher burden of cardiovascular risk factors, and presented more often with typical angina (3.2%, 19.7%, and 50.3%, p<.0001). MACE and Death/MI/Stroke events occurred in 303 and 138 patients, and event types were: all cause death (N = 64), myocardial infarction (N = 41), stroke (N = 33), late revascularization (N = 136), admission for unstable angina (N = 33), and congestive heart failure (N = 38).

**Table 1:** Demographics/Risk Factors by Risk Factor Weighted Clinical Likelihood.

**Clinical**	**All** **Patients** **N=6061**	**Very Low** **2161(36.0%)**	**Low** **2585(43.0%)**	**Moderate** **1315(22.0%)**	**P-value**
Age (years)					<0.0001*
Mean ± Std	(58.55 ± 12.07)	(52.07 ± 11.31)	(60.62 ± 10.97)	(65.13 ± 10.21)	
Sex					<0.0001*
Female	3118 (51.4%)	1660 (76.8%)	1281 (49.6%)	177 (13.5%)	
Male	2943 (48.6%)	501 (23.2%)	1304 (50.4%)	1138 (86.5%)	
Ethnicity					0.0030*
Hispanic or Latino	1567 (25.9%)	559 (25.9%)	716 (27.7%)	292 (22.2%)	
Not Hispanic or Latino	3787 (62.5%)	1376 (63.7%)	1569 (60.7%)	842 (64.0%)	
Missing	707 (11.7%)	226 (10.5%)	300 (11.6%)	181 (13.8%)	
Race					<0.0001*
Asian	139 (2.3%)	51 (2.4%)	61 (2.4%)	27 (2.1%)	
Black or African Ame	337 (5.6%)	154 (7.1%)	133 (5.1%)	50 (3.8%)	
White	5035 (83.1%)	1705 (78.9%)	2183 (84.4%)	1147 (87.2%)	
Other	550 (9.1%)	251 (11.6%)	208 (8.0%)	91 (6.9%)	
**Cardiovascular risk**					
Body Mass Index (BMI), kg/m2					0.1495
Mean ± Std	(28.49 ± 6.50)	(28.56 ± 8.03)	(28.49 ± 5.70)	(28.40 ± 5.10)	
Smoker	968 (16.0%)	325 (15.0%)	408 (15.8%)	235 (17.9%)	0.0550
Diabetes or on Diabetes Medication	1520 (25.1%)	383 (17.7%)	683 (26.4%)	454 (34.5%)	<0.0001*
Hypertension	3440 (56.8%)	900 (41.6%)	1605 (62.1%)	935 (71.1%)	<0.0001*
Dyslipidemia	3186 (52.6%)	798 (36.9%)	1516 (58.6%)	872 (66.3%)	<0.0001*
Family history positive for CAD	1926 (31.8%)	548 (25.4%)	866 (33.5%)	512 (38.9%)	<0.0001*
Atrial Fibrillation	446 (7.4%)	105 (4.9%)	225 (8.7%)	116 (8.8%)	<0.0001*
Heart Failure	272 (4.5%)	59 (2.7%)	141 (5.5%)	72 (5.5%)	<0.0001*
Peripheral artery disease (PAD)	156 (2.6%)	27 (1.2%)	69 (2.7%)	60 (4.6%)	<0.0001*
**Cardiac symptoms**					
Typical Angina	1241 (20.5%)	69 (3.2%)	510 (19.7%)	662 (50.3%)	<0.0001*
Atypical Angina	1568 (25.9%)	565 (26.1%)	740 (28.6%)	263 (20.0%)	<0.0001*
Non-cardiac Chest Pain	230 (3.8%)	121 (5.6%)	92 (3.6%)	17 (1.3%)	<0.0001*
Dyspnea	1440 (23.8%)	343 (15.9%)	701 (27.1%)	396 (30.1%)	<0.0001*
Palpitations	520 (8.6%)	254 (11.8%)	207 (8.0%)	59 (4.5%)	<0.0001*
Syncope	129 (2.1%)	67 (3.1%)	50 (1.9%)	12 (0.9%)	<0.0001*
Other symptoms	657 (10.8%)	320 (14.8%)	268 (10.4%)	69 (5.2%)	<0.0001*

### 3.2 Atherosclerosis quantification by AI-QCT

The prevalence of obstructive CAD (>50% of quantitative diameter stenosis) was 15.2% in the total cohort, and 6.8%, 15.7%. and 28.2% for very-low, low, and moderate risk, respectively (Appendix Table 1). The median total plaque volume (TPV) in the total population was 57.2 mm^3^ (25–75 IQR 16.2, 177.6) and was larger with increasing RF-CL: 25.2 mm^3^ (25–75th IQR 7.3–73.8), 68.9 mm^3^ (25–75th IQR 21.9, 184.8), and 157.8 mm^3^ (25–75th IQR 55.7, 368.1), p<.0001.

Patients were categorized according to TPV=0, <250, 250–750, and >750 mm^3^ in 7.2%, 74.9%, 15.0%, 2.9%, respectively, and according to NCPV <20, 20–125, 125–375, and >375 mm^3^ in 33.9%, 44.2%, 18.1%, and 3.7% (Fig. 1). With increasing RF-CL more patients were classified into categories with larger plaque volumes (Appendix Table 1).

**Fig. 1:**
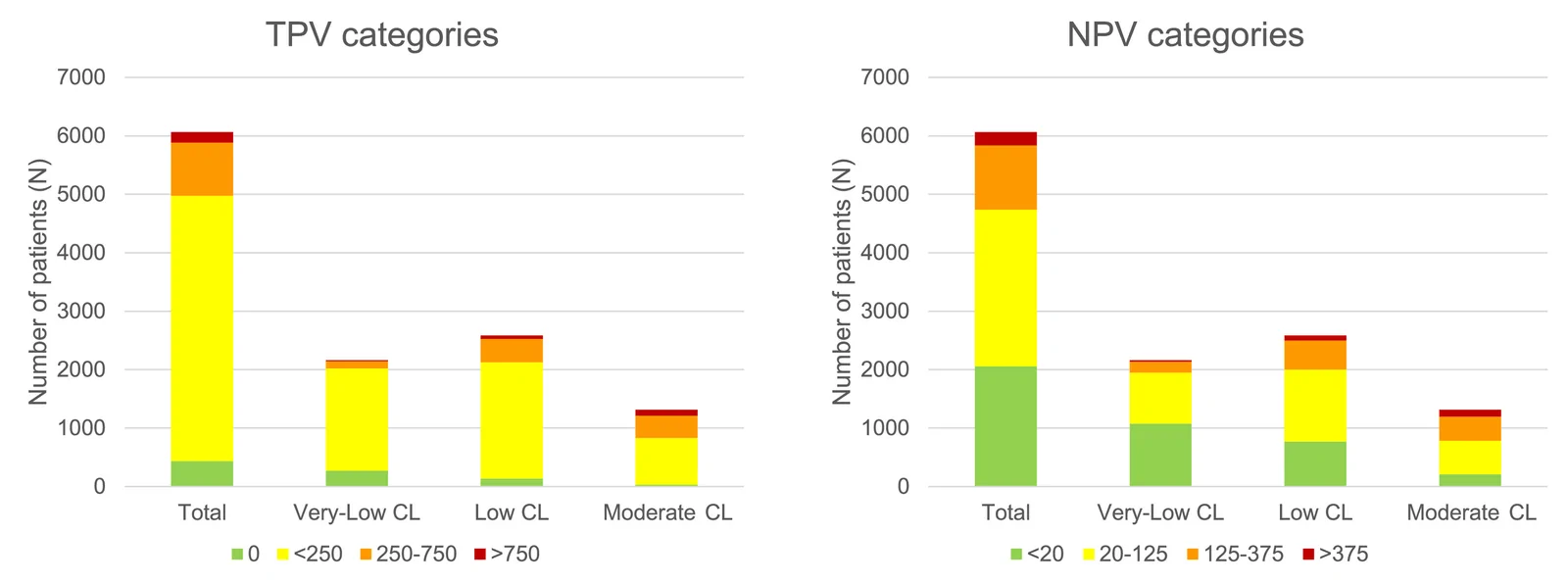
Distribution of patients in the total cohort and subdivided according to the three Risk Factor Weighted Clinical Likelihood groups.

### 3.3 Association of plaque burden categories with MACE and Death/MI/Stroke

In the total population all TPV categories were significantly associated with MACE: HR 2.5 (95% CI 1.1, 5.6), HR 10.1 (95% CI 4.4, 22.9), and HR 15.9 (95% CI 6.7, 38.0) for TPV 0–250, 250–750, and >750 mm^3^ compared with TPV=0, Table 2A. The hazard ratio of TPV 0–250 did not reach statistical significance within the very-low, and low risk RF-CL and there were no patients with TPV=0 in the ‘moderate’ category. The TPV categories >250 mm^3^ were significantly associated with MACE across all RF-CL subgroups. NCPV categorization was also significantly associated with MACE: HR 2.0 (95% CI 1.4, 2.8), HR 5.5 (95% CI 3.8, 7.8), and HR 9.4 (95% CI 6.1, 14.5) for NCPV 20–125, 125–375, and >375 mm^3^ compared with NCPV<20 mm^3^. Within the RF-CL subgroups, NCPV 20–125 vs <20 was only significant for the ‘low’ RF-CL subgroup. All NCPV categories >125 mm^3^ were significantly associated with MACE.

**Table 2A:** Hazard Ratios for the Primary Endpoint (MACE).

	Overall Patients N = 6061	Very Low 2161(36.0%)	Low 2585(43.0%)	Moderate 1315(22.0%)
Quantitative Diameter stenosis, %				
0%	Ref.	Ref.	1.07 (0.38,3.08)	0.00 (0.00,0.00)
1–24%	1.85 (0.85,4.04)	2.39 (0.72,7.93)	Ref.	Ref.
25–49%	5.49 (2.55,11.83)	5.79 (1.69,19.87)	3.14 (1.94,5.09)	2.52 (1.41,4.52)
50–69%	10.69 (4.89,23.37)	9.46 (2.37,37.86)	6.42 (3.78,10.90)	4.35 (2.36,8.02)
70–99%	18.55 (8.44,40.81)	26.46 (7.02,99.74)	12.61 (7.28,21.85)	5.69 (2.98,10.85)
100%	14.41 (6.09,34.07)	16.57 (2.77,99.21)	7.01 (2.89,17.03)	6.04 (2.89,12.66)
TPV staging system, mm^3^				
0	Ref.	Ref.	Ref.	0.00 (0.00,0.00)
>0–250	2.45 (1.09,5.55)	3.38 (0.82,13.96)	1.24 (0.45,3.38)	Ref.
>250–750	10.08 (4.44,22.92)	16.75 (3.78,74.32)	4.63 (1.67,12.82)	3.15 (2.06,4.80)
>750	15.92 (6.67,38.00)	37.35 (6.20,225.11)	6.57 (2.06,20.97)	4.91 (2.85,8.46)
NCPV Tertiles				
0–19.2	Ref.	Ref.	Ref.	Ref.
>19.2–76.2	1.38 (0.93,2.05)	1.18 (0.59,2.38)	1.37 (0.77,2.43)	1.22 (0.46,3.21)
>76.2	5.11 (3.68,7.10)	4.80 (2.64,8.74)	4.18 (2.54,6.88)	4.40 (1.93,10.05)
NCPV				
0–20	Ref.	Ref.	Ref.	Ref.
>20–125	1.97 (1.38,2.81)	1.51 (0.81,2.84)	1.94 (1.15,3.26)	1.84 (0.77,4.43)
>125–375	5.45 (3.84,7.75)	5.43 (2.73,10.80)	4.26 (2.50,7.26)	4.86 (2.09,11.33)
>375	9.38 (6.08,14.47)	17.89 (6.62,48.36)	7.26 (3.58,14.71)	6.87 (2.77,17.03)
Standard CAD classes				
<50% stenosis	Ref.	Ref.	Ref.	Ref.
1-V > 50% stenosis	4.77 (3.70,6.16)	5.78 (2.98,11.20)	5.14 (3.55,7.45)	2.77 (1.81,4.25)
2-V > 50% stenosis	4.62 (2.99,7.14)	7.37 (2.64,20.55)	4.73 (2.45,9.14)	2.63 (1.30,5.32)
3-V > 50% stenosis	8.44 (5.06,14.09)	38.96 (5.32,285.01)	4.96 (1.81,13.54)	6.14 (3.21,11.73)
Prox LAD or LM >50% stenosis				
1 vs 0	4.15 (3.18,5.41)	5.45 (2.59,11.48)	3.80 (2.53,5.70)	2.84 (1.89,4.25)
High Risk Plaque				
0	Ref.	Ref.	Ref.	Ref.
1	2.07 (1.06,4.02)	1.87 (0.26,13.49)	2.15 (0.88,5.27)	1.53 (0.48,4.83)
>1	2.79 (1.90,4.10)	1.94 (0.47,7.95)	2.08 (1.06,4.09)	2.49 (1.50,4.14)

A similar trend was observed for associations between TPV, NCPV and the endpoint Death/MI/Stroke (Table 2B).

**Table 2B:** Hazard Ratios for the Secondary Endpoint (Death/MI/Stroke).

	Overall Patients N = 6061	Very Low 2161(36.0%)	Low 2585(43.0%)	Moderate 1315(22.0%)
Quantitative Diameter stenosis, %				
0%	Ref.	Ref.	1.75 (0.49,6.19)	0.00 (0.00,0.00)
1–24%	1.59 (0.63,4.01)	2.72 (0.63,11.71)	Ref.	Ref.
25–49%	3.28 (1.30,8.31)	6.63 (1.48,29.63)	2.09 (0.97,4.52)	1.70 (0.78,3.69)
50–69%	6.89 (2.67,17.80)	7.08 (1.18,42.39)	7.45 (3.52,15.75)	2.47 (1.05,5.82)
70–99%	6.65 (2.42,18.30)	13.82 (2.31,82.71)	3.86 (1.24,11.96)	3.26 (1.31,8.11)
100%	6.62 (2.10,20.87)	25.43 (3.58,180.66)	9.75 (3.14,30.23)	0.71 (0.09,5.53)
TPV staging system, mm3				
0	Ref.	Ref.	Ref.	0.00 (0.00,0.00)
>0–250	1.85 (0.68,5.07)	4.99 (0.68,36.57)	0.64 (0.19,2.09)	Ref.
>250–750	6.01 (2.16,16.68)	18.05 (2.22,146.93)	2.30 (0.68,7.76)	2.53 (1.35,4.75)
>750	7.36 (2.34,23.13)	51.22 (4.60,569.87)	2.48 (0.50,12.27)	2.77 (1.10,6.98)
NCP Tertiles				
0–19.2	Ref.	Ref.	Ref.	Ref.
>19.2–76.2	0.75 (0.43,1.29)	0.76 (0.33,1.78)	0.93 (0.38,2.29)	0.45 (0.12,1.66)
>76.2	3.04 (2.00,4.60)	3.20 (1.60,6.41)	3.45 (1.66,7.21)	2.02 (0.79,5.15)
NCPV				
0–20	Ref.	Ref.	Ref.	Ref.
>20–125	1.29 (0.82,2.03)	1.06 (0.51,2.20)	1.67 (0.77,3.62)	0.94 (0.34,2.65)
>125–375	2.99 (1.88,4.75)	3.26 (1.39,7.64)	3.36 (1.51,7.49)	2.02 (0.75,5.41)
>375	4.54 (2.40,8.55)	11.34 (3.29,39.06)	3.30 (0.89,12.19)	2.95 (0.97,9.03)
Standard CAD classes				
<50% stenosis	Ref.	Ref.	Ref.	Ref.
1-V > 50% stenosis	3.43 (2.32,5.07)	4.09 (1.71,9.79)	4.93 (2.76,8.81)	1.68 (0.84,3.38)
2-V > 50% stenosis	4.07 (2.18,7.60)	4.70 (1.13,19.62)	4.50 (1.59,12.75)	2.76 (1.06,7.14)
3-V > 50% stenosis	0.87 (0.12,6.22)	0.00 (0.00,.)	0.00 (0.00,.)	0.93 (0.13,6.81)
Prox LAD or LM >50% stenosis	Ref.	Ref.	Ref.	Ref.
1 vs 0	2.63 (1.69,4.08)	1.70 (0.41,7.06)	3.31 (1.71,6.44)	1.88 (0.95,3.72)
High Risk Plaque				
0	Ref.	Ref.	Ref.	Ref.
1	1.40 (0.45,4.40)	0.00 (0.00, 0.00)	0.98 (0.14,7.10)	2.35 (0.57,9.74)
>1	1.34 (0.62,2.86)	0.00 (0.00, 0.00)	0.52 (0.07,3.76)	1.90 (0.80,4.49)

Kaplan Meier curves are provided for the TPV and NCPV stages for both endpoints, Fig. 2A and 2B, showing most increase in risk after TPV>250 mm^3^, and NCPV 125 mm^3^.

**Fig. 2A:**
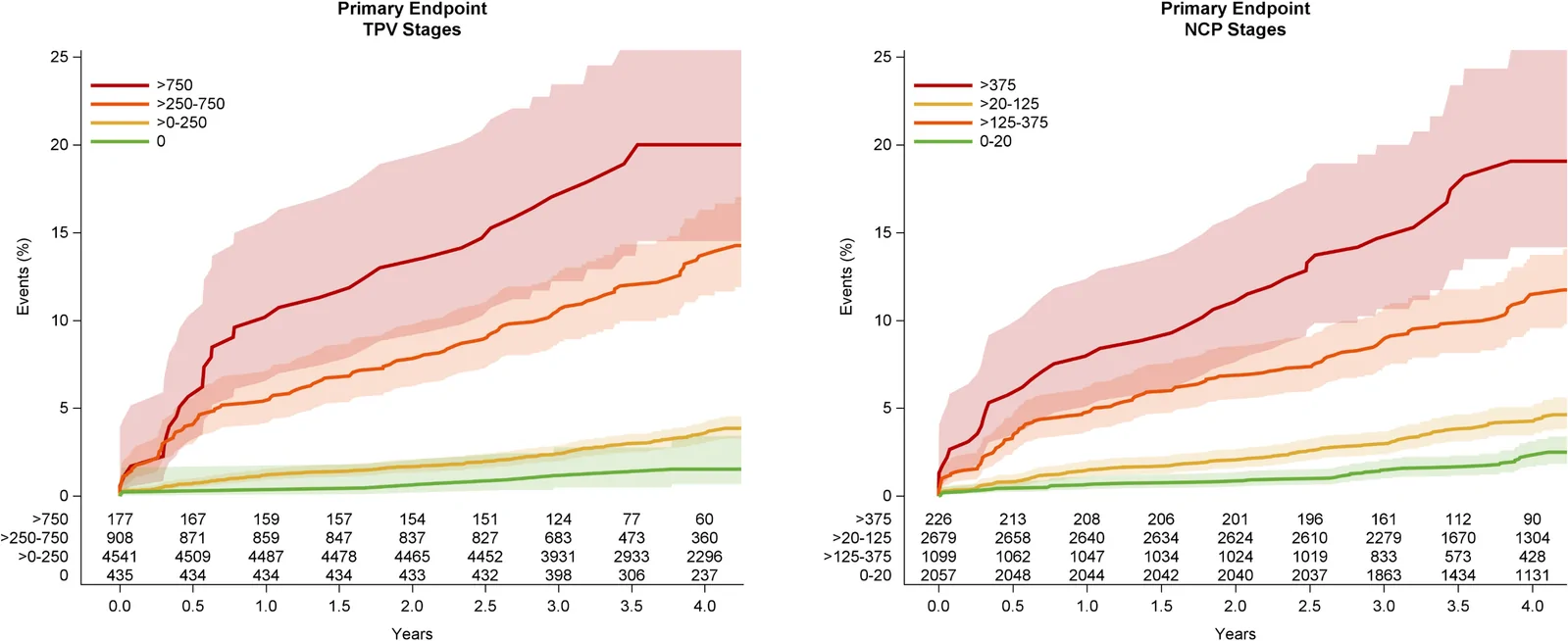
Kaplan Meier curves with 95% CI’s for the primary endpoint (MACE) according tot eh total plaque volume (TPV) stages and the noncalcified plaque (NCP) stages.

**Fig. 2B:**
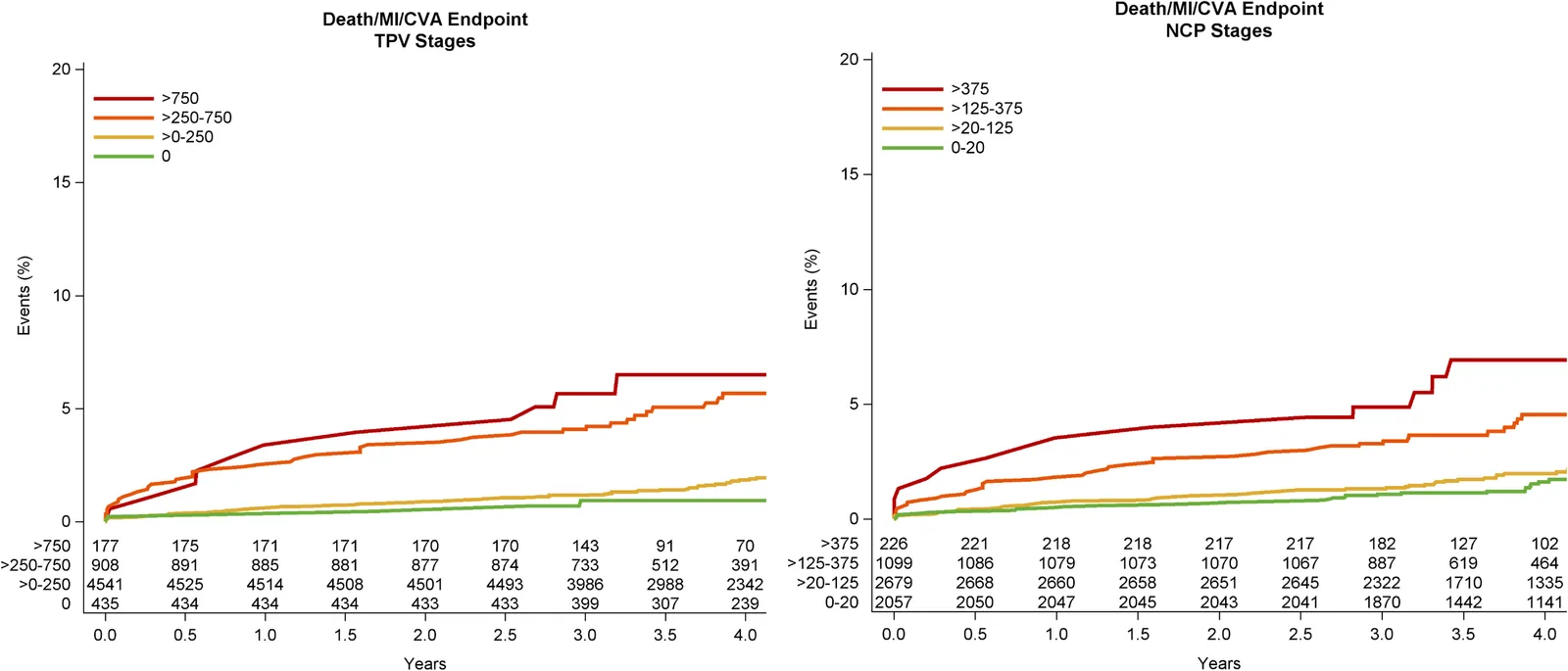
Kaplan Meier curves for the secondary endpoint (Death/MI/Stroke) according to the total plaque volume (TPV) stages and the noncalcified plaque (NCP) stages.

### 3.4 Absolute MACE and Death/MI/Stroke according to plaque burden

The 4-year MACE rates according to the TPV stages were 1.4%, 3.3%, 12.6%, and 18.6% for TPV=0, 0–250, 250–750, and >750 mm^3^, Table 3A. The event rates across the RF-CL spectrum were comparable within the specific TPV categories. According to RF-CL very-low, low, and moderate, the MACE rates were: 0.7, 2.9, 0.0% for TPV=0 mm^3^; 2.3%, 3.6%, 4.6% for TPV 0–250 mm^3^; 10.3%, 12.3%, 13.5% for TPV 250–750 mm^3^; and 18.8%, 16.7%, and 19.8% for TPV>750 mm^3^, Fig. 3A/Appendix Fig. 2. According to RF-CL very-low, low, and moderate, the MACE rates were: 1.7, 2.5, 2.9% for NPV 0–20 mm^3^; 2.4%, 4.6%, 5.2% for NPV 20–125 mm^3^; 7.8%, 9.4%, 12.7% for NPV 125–375 mm^3^; and 22.7%, 15.3%, and 17.6% for NCPV>375 mm^3^, Fig. 3A. Results for the Death/MI/Stroke endpoint are provided in Table 3B and Fig. 3B; and show a similar pattern as with the MACE endpoint.

**Table 3A:** 4-Year Event Rates for the Primary Endpoint (MACE).

	Overall Patients N = 6061	Very Low 2161(36.0%)	Low 2585(43.0%)	Moderate 1315(22.0%)
Quantitative Diameter stenosis, %				
0%	7/580 (1.2%)	3/362 (0.8%)	4/182 (2.2%)	0/36 (0.0%)
1–24%	67/3043 (2.2%)	24/1281 (1.9%)	26/1265 (2.1%)	17/497 (3.4%)
25–49%	95/1514 (6.3%)	16/371 (4.3%)	45/732 (6.1%)	34/411 (8.3%)
50–69%	61/526 (11.6%)	6/88 (6.8%)	29/244 (11.9%)	26/194 (13.4%)
70–99%	53/272 (19.5%)	8/43 (18.6%)	25/116 (21.6%)	20/113 (17.7%)
100%	20/126 (15.9%)	2/16 (12.5%)	6/46 (13.0%)	12/64 (18.8%)
TPV staging system, mm3 (with 95% CI’s)				
0	6/435 (1.4%) (0.6 - 3.0)	2/270 (0.7%) (0.2 - 2.7)	4/136 (2.9%) (1.1 - 7.3)	0/29 (0.0%) (0.0 - 0.0)
>0–250	150/4541 (3.3%) (2.8 - 3.9)	41/1749 (2.3%) (1.7 - 3.2)	72/1992 (3.6%) (2.9 - 4.5)	37/800 (4.6%) (3.4 - 6.3)
>250–750	114/908 (12.6%) (10.6 - 14.9)	13/126 (10.3%) (6.1 - 16.9)	49/397 (12.3%) (9.5 - 15.9)	52/385 (13.5%) (10.5 - 17.3)
>750	33/177 (18.6%) (13.6 - 25.0)	3/16 (18.8%) (6.6 - 43.0)	10/60 (16.7%) (9.3 - 28.0)	20/101 (19.8%) (13.2 - 28.6)
NCPV Tertiles				
0–19.2	43/2020 (2.1%)	18/1059 (1.7%)	19/757 (2.5%)	6/204 (2.9%)
>19.2–76.2	58/2022 (2.9%)	14/729 (1.9%)	31/929 (3.3%)	13/364 (3.6%)
>76.2	202/2019 (10.0%)	27/373 (7.2%)	85/899 (9.5%)	90/747 (12.0%)
NCPV, mm3 (with 95% CI’s)				
0–20	43/2057 (2.1%) (1.6 - 2.8)	18/1075 (1.7%) (1.1 - 2.6)	19/772 (2.5%) (1.6 - 3.8)	6/210 (2.9%) (1.3 - 6.1)
>20–125	107/2679 (4.0%) (3.3 - 4.8)	21/872 (2.4%) (1.6 - 3.7)	56/1230 (4.6%) (3.5 - 5.9)	30/577 (5.2%) (3.7 - 7.3)
>125–375	114/1099 (10.4%) (8.7 - 12.3)	15/192 (7.8%) (4.8 - 12.5)	47/498 (9.4%) (7.2 - 12.3)	52/409 (12.7%) (9.8 - 16.3)
>375	39/226 (17.3%) (12.9 - 22.7)	5/22 (22.7%) (10.1 - 43.4)	13/85 (15.3%) (9.2 - 24.4)	21/119 (17.6%) (11.8 - 25.5)
Standard CAD classes				
<50% stenosis	175/5225 (3.3%)	43/2034 (2.1%)	77/2215 (3.5%)	55/976 (5.6%)
1-V > 50% stenosis	89/606 (14.7%)	11/95 (11.6%)	44/275 (16.0%)	34/236 (14.4%)
2-V > 50% stenosis	23/164 (14.0%)	4/30 (13.3%)	10/69 (14.5%)	9/65 (13.8%)
3-V > 50% stenosis	16/66 (24.2%)	1/2 (50.0%)	4/26 (15.4%)	11/38 (28.9%)
Prox LAD or LM >50% stenosis	72/461 (15.6%)	8/67 (11.9%)	30/199 (15.1%)	34/195 (17.4%)
High Risk Plaque				
0	265/5725 (4.6%)	56/2098 (2.7%)	121/2440 (5.0%)	88/1187 (7.4%)
1	9/100 (9.0%)	1/22 (4.5%)	5/51 (9.8%)	3/27 (11.1%)
>1	29/236 (12.3%)	2/41 (4.9%)	9/94 (9.6%)	18/101 (17.8%)

**Figure:**
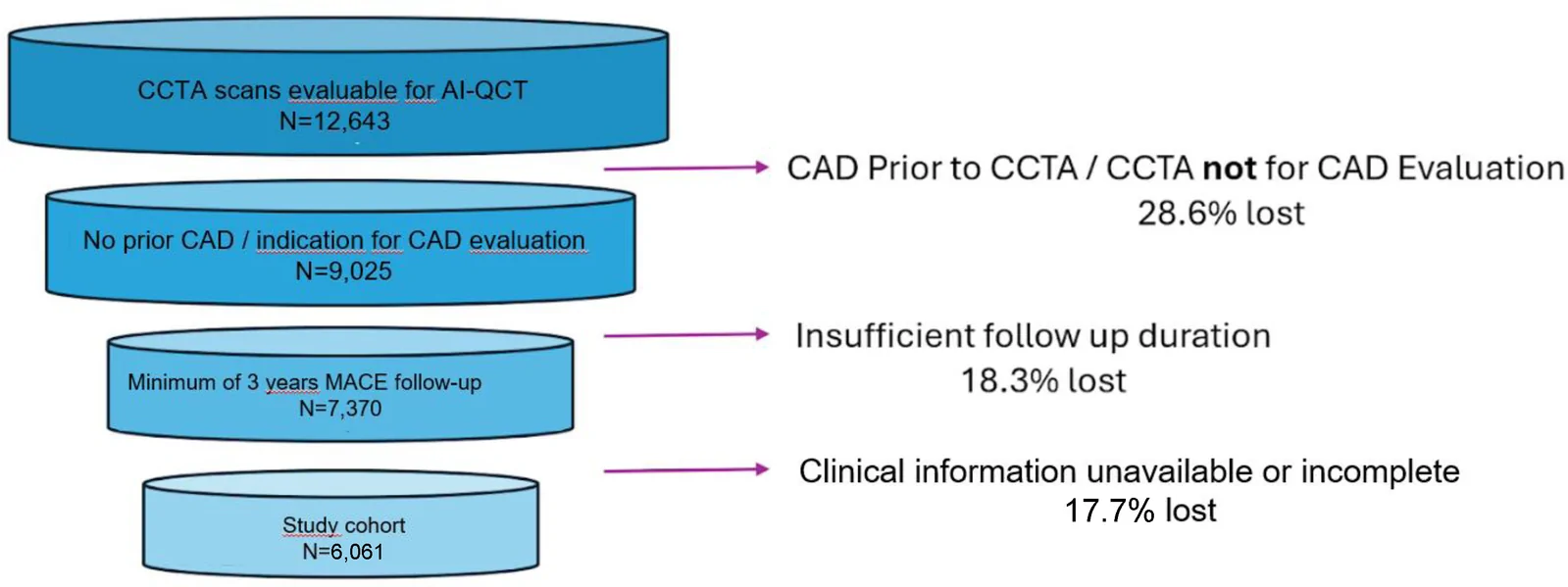
Fig. A1.

**Fig. 3A:**
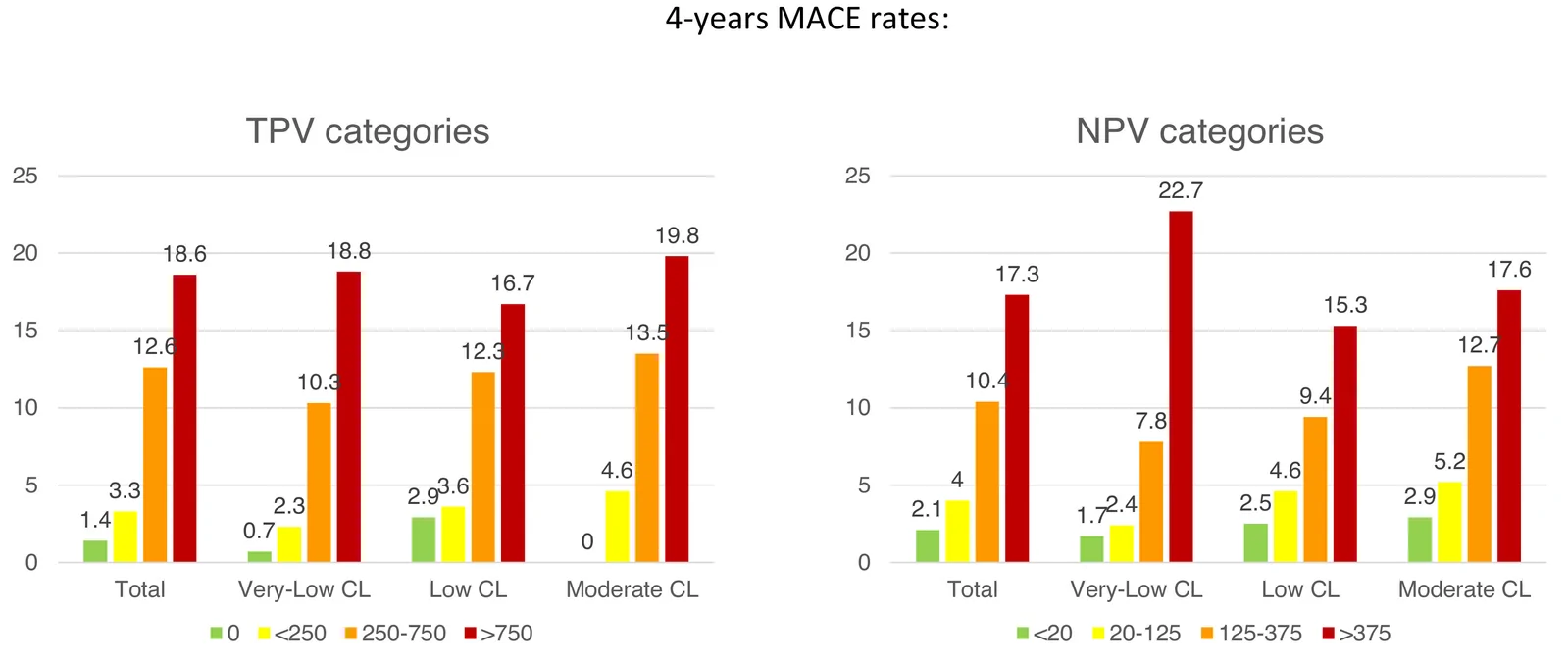
4 year MACE rates according to the total plaque volume (TPV) and the noncalcified plaque (NCP) staging systems.

**Table 3B:** 4-Year Event Rates for the Secondary Endpoint (Death/MI/Stroke).

	All Patients N = 6061	Very Low 2161(36.0%)	Low 2585(43.0%)	Moderate 1315(22.0%)
Quantitative Diameter stenosis, %				
0%	5/580 (0.9%)	2/362 (0.6%)	3/182 (1.6%)	0/36 (0.0%)
1–24%	41/3043 (1.3%)	18/1281 (1.4%)	12/1265 (0.9%)	11/497 (2.2%)
25–49%	41/1514 (2.7%)	12/371 (3.2%)	14/732 (1.9%)	15/411 (3.6%)
50–69%	29/526 (5.5%)	3/88 (3.4%)	16/244 (6.6%)	10/194 (5.2%)
70–99%	15/272 (5.5%)	3/43 (7.0%)	4/116 (3.4%)	8/113 (7.1%)
100%	7/126 (5.6%)	2/16 (12.5%)	4/46 (8.7%)	1/64 (1.6%)
TPV staging system, mm3 (with 95% CI’s)				
0	4/435 (0.9%) (0.4 - 2.3)	1/270 (0.4%) (0.1 - 2.1)	3/136 (2.2%) (0.8 - 6.3)	0/29 (0.0%) (0.0 - 0.0)
>0–250	76/4541 (1.7%) (1.3 - 2.1)	30/1749 (1.7%) (1.2 - 2.4)	28/1992 (1.4%) (1.0 - 2.0)	18/800 (2.3%) (1.4 - 3.5)
>250–750	47/908 (5.2%) (3.9 - 6.8)	7/126 (5.6%) (2.7 - 11.0)	19/397 (4.8%) (3.1 - 7.4)	21/385 (5.5%) (3.6 - 8.2)
>750	11/177 (6.2%) (3.5 - 10.8)	2/16 (12.5%) (3.5 - 36.0)	3/60 (5.0%) (1.7 - 13.7)	6/101 (5.9%) (2.8 - 12.4)
NCPV Tertiles				
0–19.2	30/2020 (1.5%)	16/1059 (1.5%)	9/757 (1.2%)	5/204 (2.5%)
>19.2–76.2	22/2022 (1.1%)	8/729 (1.1%)	10/929 (1.1%)	4/364 (1.1%)
>76.2	86/2019 (4.3%)	16/373 (4.3%)	34/899 (3.8%)	36/747 (4.8%)
NCPV, mm3 (with 95% CI’s)				
0–20	30/2057 (1.5%) (1.0 - 2.1)	16/1075 (1.5%) (0.9 - 2.4)	9/772 (1.2%) (0.6 - 2.2)	5/210 (2.4%) (1.0 - 5.5)
>20–125	49/2679 (1.8%) (1.4 - 2.4)	13/872 (1.5%) (0.9 - 2.5)	23/1230 (1.9%) (1.2 - 2.8)	13/577 (2.3%) (1.3 - 3.8)
>125–375	45/1099 (4.1%) (3.1 - 5.4)	8/192 (4.2%) (2.1 - 8.0)	18/498 (3.6%) (2.3 - 5.6)	19/409 (4.6%) (3.0 - 7.1)
>375	14/226 (6.2%) (3.7 - 10.1)	3/22 (13.6%) (4.7 - 33.3)	3/85 (3.5%) (1.2 - 9.9)	8/119 (6.7%) (3.4 - 12.7)
Standard CAD classes				
<50% stenosis	91/5225 (1.7%)	32/2034 (1.6%)	31/2215 (1.4%)	28/976 (2.9%)
1-V > 50% stenosis	35/606 (5.8%)	6/95 (6.3%)	18/275 (6.5%)	11/236 (4.7%)
2-V > 50% stenosis	11/164 (6.7%)	2/30 (6.7%)	4/69 (5.8%)	5/65 (7.7%)
3-V > 50% stenosis	1/66 (1.5%)	0/2 (0.0%)	0/26 (0.0%)	1/38 (2.6%)
Prox LAD or LM >50% stenosis	24/461 (5.2%)	2/67 (3.0%)	11/199 (5.5%)	11/195 (5.6%)
High Risk Plaque				
0	128/5725 (2.2%)	40/2098 (1.9%)	51/2440 (2.1%)	37/1187 (3.1%)
1	3/100 (3.0%)	0/22 (0.0%)	1/51 (2.0%)	2/27 (7.4%)
>1	7/236 (3.0%)	0/41 (0.0%)	1/94 (1.1%)	6/101 (5.9%)

**Fig. 3B:**
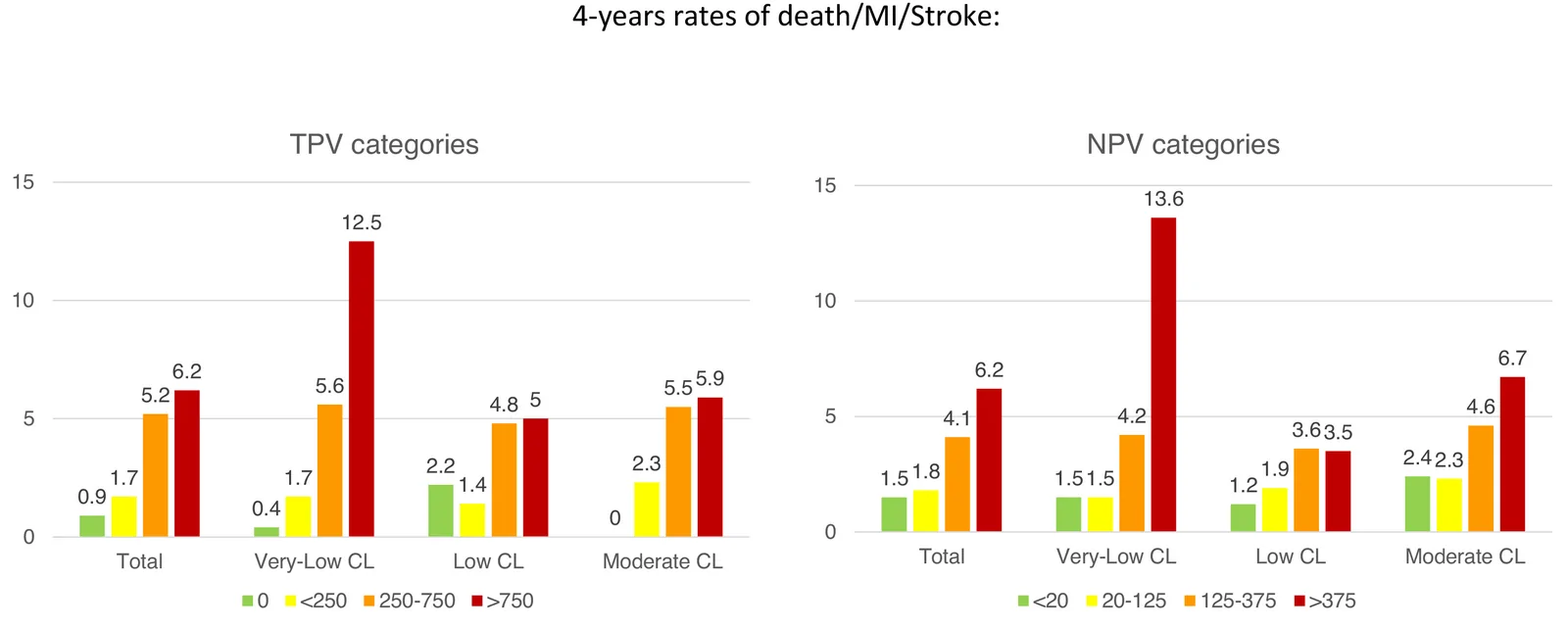
4 year rates of Death/MI/Stroke according to the total plaque volume (TPV) and the noncalcified plaque (NCP) staging systems.

### 3.5 Plaque burden and stenosis combined for risk stratification

Classifying patients as low risk by the presence of TPV <250 mm^3^ and maximal diameter stenosis <30% resulted in a larger proportion of low-risk patients (65.7%) compared with using TPV=0 (7.2%) or NCPV<20 m^3^ (33.9%) as treshold (Appendix Fig. 3s). Patients with either >30% stenosis or TPV >250 were at increased risk compared with those with TPV <250 mm^3^ and stenosis <30%: HR 2.94 (95% CI 2.2, 4.0) for MACE and HR 2.0 (95% CI 1.3, 3.1) for Death/MI/stroke, Table 4A, Table 4B. Fig. 4 shows consistent 4-year rates of MACE and Death/MI/stroke for the three risk groups across the RF-CL categories.

**Table 4A:** Hazard Ratios for the Primary Endpoint (MACE).

	**All** **Patients** **N=6061**	**Very Low** **2161(36.0%)**	**Low** **2585(43.0%)**	**Moderate** **1315(22.0%)**
Stenosis and TPV				
<30% stenosis and TPV<250	Ref.	Ref.	Ref.	Ref.
≥30% stenosis or TPV≥250	2.94 (2.15,4.01)	2.34 (1.18,4.65)	3.05 (1.95,4.79)	2.40 (1.31,4.40)
≥30% stenosis and TPV≥250	7.17 (5.51,9.34)	8.31 (4.55,15.17)	6.86 (4.57,10.28)	5.12 (3.08,8.53)

**Table 4B:** Hazard Ratios for the Secondary Endpoint (Death/MI/Stroke).

	**All** **Patients** **N=6061**	**Very Low** **2161(36.0%)**	**Low** **2585(43.0%)**	**Moderate** **1315(22.0%)**
Stenosis and TPV				
<30% stenosis and TPV<250	Ref.	Ref.	Ref.	Ref.
≥30% stenosis or TPV≥250	1.97 (1.25,3.09)	1.63 (0.67,3.97)	2.28 (1.12,4.65)	1.76 (0.75,4.16)
≥30% stenosis and TPV≥250	4.41 (3.03,6.43)	5.91 (2.75,12.70)	5.28 (2.83,9.85)	3.00 (1.47,6.13)

**Fig. 4:**
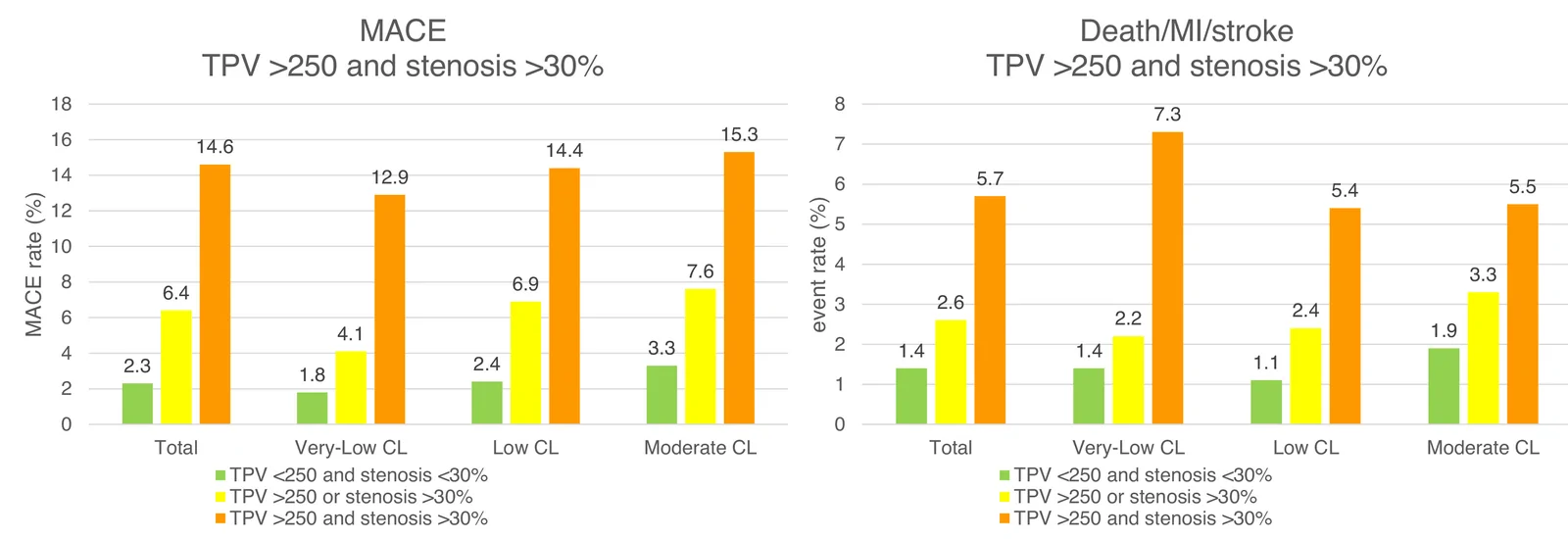
Rates of 4-year of the primary and secondary endpoint according to the three Risk Factor Weighted Clinical Likelihood groups.

### 3.6 Multivariable analysis adjusted for clinical risk factors and quantitative stenosis severity

There was little effect modification of the hazard ratios for the TPV and NCPV staging systems predicting the primary endpoint when adjusting for age, sex, diabetes, hypertension, dyslipidemia and smoking (Table 5A). Further adjustment for quantitative stenosis severity lowered the hazard ratios, but plaque burden remained a significant predictor in many subgroups (Table 5B).

**Table 5A:** Hazard ratios for the primary endpoint (MACE), with adjustment.

	Overall Patients N = 6061	Very Low 2161(36.0%)	Low 2585(43.0%)	Moderate 1315(22.0%)
TPV staging system, mm^3^ (adjusted for age, sex, diabetes, hypertension, dyslipidemia, smoking)				
0	Ref.	Ref.	Ref.	0.00 (0.00,0.00)
>0–250	2.99 (1.10, 8.10)	2.80 (0.67, 11.66)	2.04 (0.50, 8.37)	Ref.
>250–750	10.96 (3.97, 30.25)	10.94 (2.36, 50.70)	8.23 (1.98, 34.20)	2.92 (1.86, 4.57)
>750	13.57 (4.62, 39.81)	21.76 (3.40, 139.29)	8.41 (1.74, 40.73)	4.46 (2.43, 8.18)
TPV staging system, mm^3^ (adjusted for age, sex, diabetes, hypertension, dyslipidemia, smoking and quantitative diameter stenosis (0%, 1–24%, 25–49%, 50–69%, 70–99%, 100%))				
0	Ref.	Ref.	Ref.	0.00 (0.00,0.00)
>0–250	1.65 (0.60, 4.55)	1.47 (0.34, 6.47)	0.94 (0.22, 3.99)	Ref.
>250–750	3.63 (1.25, 10.56)	2.93 (0.53, 16.23)	2.11 (0.46, 9.65)	2.08 (1.26, 3.44)
>750	3.36 (1.06, 10.63)	4.38 (0.55, 35.02)	1.39 (0.25, 7.76)	2.67 (1.33, 5.33)
				
NCPV (adjusted for age, sex, diabetes, hypertension, dyslipidemia, smoking)				
0–20	Ref.	Ref.	Ref.	Ref.
>20–125	1.88 (1.28, 2.76)	1.30 (0.68, 2.46)	2.32 (1.25, 4.28)	1.90 (0.78, 4.59)
>125–375	4.64 (3.10, 6.93)	3.76 (1.79, 7.90)	5.37 (2.84, 10.16)	4.48 (1.88, 10.66)
>375	7.20 (4.35, 11.93)	12.57 (4.46, 35.46)	7.95 (3.44, 18.37)	6.50 (2.53, 16.71)
NCPV (adjusted for age, sex, diabetes, hypertension, dyslipidemia, smoking and quantitative diameter stenosis (0%, 1–24%, 25–49%, 50–69%, 70–99%, 100%))				
0–20	Ref.	Ref.	Ref.	Ref.
>20–125	1.37 (0.92, 2.03)	0.90 (0.46, 1.76)	1.57 (0.83, 2.96)	1.48 (0.60, 3.64)
>125–375	2.17 (1.37, 3.44)	1.60 (0.66, 3.85)	2.21 (1.07, 4.54)	2.52 (0.97, 6.52)
>375	2.44 (1.35, 4.41)	4.41 (1.38, 14.14)	1.94 (0.73, 5.19)	3.07 (1.06, 8.93)

## 4 Discussion

Prior studies quantifying coronary atheroma burden using intravascular ultrasound have demonstrated a strong association with future major cardiovascular events [19,20]. The invasive nature of these techniques and their inability to capture atherosclerosis in all coronary arteries and side branches limit their utility for risk assessment in the majority of patients with suspected CAD. Comprehensive assessment of total coronary atherosclerosis has become feasible through AI-guided, semi-automated quantitative evaluation of coronary CT angiography (CCTA). Total plaque volume (TPV) measured by coronary CT has emerged as a key marker for identifying early cardiovascular risk and may allow detection before the onset of obstructive CAD. In the ISCHEMIA trial, which included high-risk patients with obstructive CAD as a minimum criterion, TPV clearly stratified future MACE events [21].

The CONFIRM 2 registry represents a global cohort of patients typically referred for CCTA, who generally have a lower pretest risk profile. The aim of the present study was to evaluate the ability of quantitative atherosclerosis assessment to stratify cardiovascular risk in a large population and to provide absolute event rates associated with varying plaque volumes. Specifically, we analyzed patients categorized into very-low, low, and moderate likelihood of obstructive CAD as defined by the ESC guidelines, based on age, sex, risk factors, and symptom typicality.

The main observation was that event risk was driven by plaque burden rather than by the RF-CL likelihood for obstructive CAD. Absolute event rates were consistently low among patients with no or minimal atherosclerosis, increased proportionally with plaque severity, and were consistent across the very-low, low, and moderate RF-CL categories . Four-year MACE rates for TPV = 0, 0–250 mm³, 250–750 mm³, and > 750 mm³ averaged 1%, 3%, 12%, and 19%, respectively, irrespective of RF-CL classification. A second key finding was that risk increased exponentially for both endpoints when TPV exceeded 250 mm³ and when non-calcified plaque (NCPV) volume exceeded 125 mm³. Extrapolating these four-year findings to ten-year event rates would classify patients with TPV > 250 mm³ or NCPV > 125 mm³ as high risk according to the SCORE2 algorithm, given their estimated > 10% cardiovascular event rate (assuming that 50% of deaths in the current study were cardiovascular [22,23].

These findings can be placed in context with prior studies demonstrating the prognostic value of quantitatively assessed plaque burden in large multicenter cohorts [24]. Using data from the SCOT-HEART trial, Lin et al. identified an optimal TPV threshold of 238.5 mm³ by the Youdens Index for prediction of myocardial infarction, associated with a univariable hazard ratio of 7.3 (95% CI, 2.6–16.5). Data from the PROMISE trial showed an optimal prognostic cut off of TPV to be 87 mm^3^ wich was independent from clinical factors and qualitative CCTA findings [25]. Among 3711 patients in the ISCHEMIA trial, Nurmohamed et al. reported a univariable hazard ratio of 1.6 (95% CI, 1.3–2.0) per quartile increase in TPV (559 mm³) for prediction of the primary outcome. The current study extends these findings by providing insights into TPV thresholds at which risk increases, along with absolute event rates across intermediate-term follow-up.

The prevalence of obstructive CAD was in line with expectations according to the very-low, low, and moderate RF-CL categories: 6.8%, 15.7%, and 28.2%, respectively. When considering NCPV 〈 20 mm³ as potential CT noise rather than true plaque, 50.3%, 70.1%, and 84.0% of patients nonetheless demonstrated atherosclerosis by AI-QCT. As previously shown, AI-QCT identifies a larger proportion of patients with plaque due to its ability to detect very small volumes. In the population-based MiHEART study, 63.3% of male participants were classified as having plaque by visual assessment, whereas 81.5% were identified by AI-QCT as having 〉 20 mm³ of plaque [26]. The prognostic importance of TPV < 250 mm³ and NCPVV < 125 mm³ remains uncertain. The hazard ratio for these subgroups was approximately 2.0 for the MACE endpoint and 1.9 for the composite of death, myocardial infarction, or stroke, although the latter did not reach statistical significance. This represents an important subgroup, as a large proportion of patients were classified within these ranges. When TPV < 250 mm³ was combined with additional low-risk features, such as maximal stenosis < 30%, event rates were only slightly higher than those with TPV = 0 or NCPVV < 20 mm³. Approximately two-thirds of the population could be categorized within this low-burden group, a category that may be used for de-risking patients with minimal atherosclerosis. Given the exponential association between plaque burden and cardiovascular risk, increasing treatment intensity in proportion to plaque volume could be justified. However, caution is warranted, as small plaque volumes carry limited risk over intermediate-term follow-up. The relatively short follow-up duration of the current study must also be acknowledged, as hard events generally accrue over longer periods. Early detection of atherosclerosis may enable anti-atherosclerotic interventions that promote primordial prevention and stabilization of the atherosclerotic disease process [27].

The current findings confirm earlier large CT registries that evaluated atherosclerosis and stenosis by visual estimation and demonstrated that the extent of atherosclerosis, irrespective of stenosis, drives risk. With automated quantification, especially of the non-calcified plaque component, atherosclerotic burden can now be measured objectively—something not achievable through visual evaluation. Previous studies have shown that non-calcified plaque burden is a stronger predictor of events than the calcified component, as seen with calcium scoring [8,11]. Finally, these findings support the Lancet Commission’s call to rethink CAD, shifting the paradigm from ischemia to atheroma [28]. Ongoing randomized trials—SCOT-HEART 2 (NCT03920176), DANE-HEART (NCT05677386), and TRANSFORM (NCT06112418)—will further evaluate treatment strategies guided by CT-quantified atherosclerosis.

### 4.1 Limitations

This study has several limitations. The population reflects symptomatic patients referred for coronary CT, characterized by a low overall event rate and correspondingly wider confidence intervals for effect estimates. The distribution of patients across plaque volume categories was comparable to that of a population-based (asymptomatic) cohort [26]. The four-year follow-up duration may have limited the prognostic accuracy for patients with low plaque burden, as hard events typically accrue over longer time horizons. Risk estimates may have been attenuated by treatment initiation following the CT scan. The incremental prognostic value of individual high-risk plaque features was not evaluated. Plaque thresholds associated with significant risk may not be directly generalizable to other software platforms with different quantification algorithms. Finally, cause-specific mortality data were not available. Tube voltage and reconstruction kernels differed between patients, which may have affected plaque composition types.

## 5 Conclusion

Among a large cohort of symptomatic patients evaluated by coronary CT angiography, the burden of atherosclerosis quantified by AI-QCT was the principal driver of cardiovascular events. Event rates increased progressively with plaque volume stages and were consistent across the clinical likelihood subgroups for obstructive CAD. While quantitative atherosclerosis was present in the majority of patients, risk increased significantly from 250 mm^3^ of TPV and 125 mm^3^ of NCPV.

## Central illustration

**Figure:**
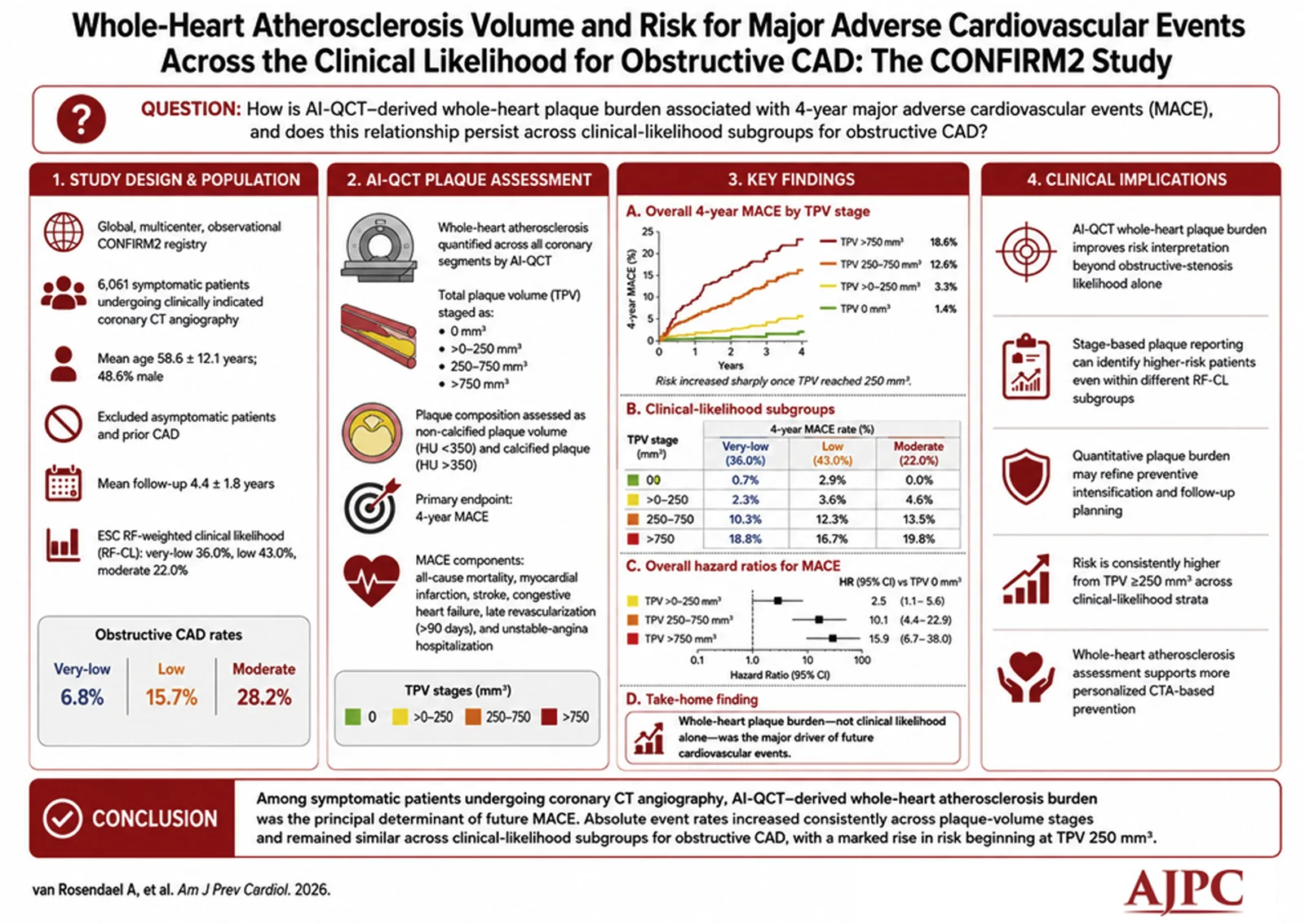

## Declaration of generative AI and AI-assisted technologies in the manuscript preparation process

During the preparation of this work, the authors used ChatGPT for the creation of the central illustration. The authors reviewed and edited the output as needed and take full responsibility for the content of the published article

## CRediT authorship contribution statement

**Alexander van Rosendael:** Writing – review & editing, Writing – original draft, Methodology, Investigation, Formal analysis, Data curation, Conceptualization. **Rine Nakanishi:** Writing – review & editing. **Jeroen J. Bax:** Writing – review & editing. **Gianluca Pontone:** Writing – review & editing. **Saima Mushtaq:** Writing – review & editing. **Ronny R. Buechel:** Writing – review & editing. **Christoph Gräni:** Writing – review & editing. **Gudrun Feuchtner:** Writing – review & editing. **Pietro G. Lacaita:** Writing – review & editing. **Amit R. Patel:** Writing – review & editing. **Cristiane C. Singulane:** Writing – review & editing. **Andrew D. Choi:** Writing – review & editing. **Mouaz Al-Mallah:** Writing – review & editing. **Daniele Andreini:** Writing – review & editing. **Ronald P. Karlsberg:** Writing – review & editing. **Geoffrey Cho:** Writing – review & editing. **Carlos E. Rochitte:** Writing – review & editing. **Mirvat Alasnag:** Writing – review & editing. **Ashraf Hamdan:** Writing – review & editing. **Filippo Cademartiri:** Writing – review & editing. **Erica Maffei:** Writing – review & editing. **Hugo Marques:** Writing – review & editing. **Pedro M. Gonçalves Pereira:** Writing – review & editing. **Himanshu Gupta:** Writing – review & editing. **Martin Hadamitzky:** Writing – review & editing. **Omar Khalique:** Writing – review & editing. **Dinesh Kalra:** Writing – review & editing. **James D. Mills:** Writing – review & editing. **Nick S. Nurmohamed:** Writing – review & editing. **Paul Knaapen:** Writing – review & editing. **Matthew Budoff:** Writing – review & editing. **Kashif Shaikh:** Writing – review & editing. **Enrico Martin:** Writing – review & editing. **David M. German:** Writing – review & editing. **Maros Ferencik:** Writing – review & editing. **Andrew C. Oehler:** Writing – review & editing. **Roderick Deaño:** Writing – review & editing. **Prashant Nagpal:** Writing – review & editing. **Marly van Assen:** Writing – review & editing. **Carlo Nicola De Cecco:** Writing – review & editing. **Vasileios Kamperidis:** Writing – review & editing. **Borek Foldyna:** Writing – review & editing. **Jan Michael Brendel:** Writing – review & editing. **Victor Y. Cheng:** Writing – review & editing. **Kelley Branch:** Writing – review & editing. **Marcio Bittencourt:** Writing – review & editing. **Sabha Bhatti:** Writing – review & editing. **Venkateshwar Polsani:** Writing – review & editing. **George Wesbey:** Writing – review & editing. **Rhanderson Cardoso:** Writing – review & editing. **Ron Blankstein:** Writing – review & editing. **Augustin Delago:** Writing – review & editing. **Amit Pursnani:** Writing – review & editing. **Amro Alsaid:** Writing – review & editing. **Vasvi Singh:** Writing – review & editing. **Melissa Aquino:** Writing – review & editing. **Ibrahim Danad:** Writing – review & editing, Writing – original draft, Validation, Project administration, Methodology, Conceptualization.

## Declaration of competing interest

The authors declare that they have no known competing financial interests or personal relationships that could have appeared to influence the work reported in this paper.

## Figures and Tables

**Fig. A2:**
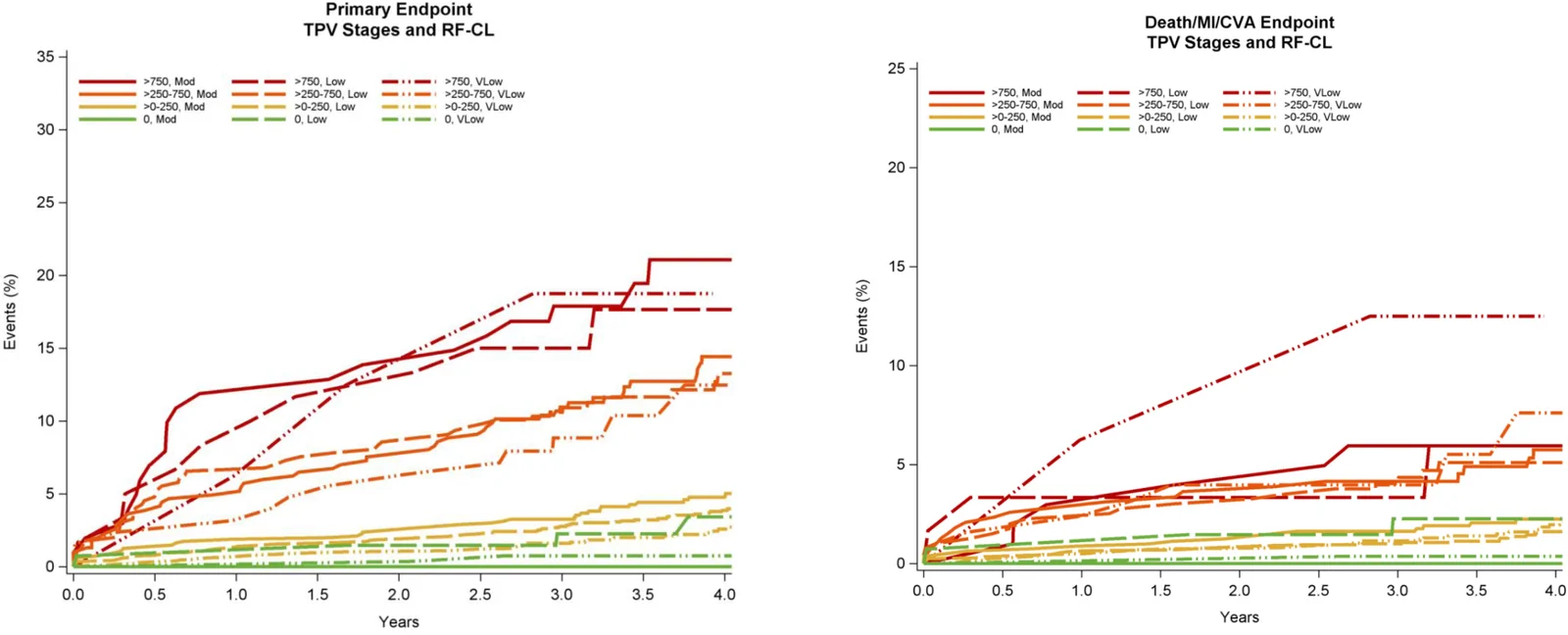
Kaplan Meier curves for the primary endpoint (MACE) according to the total plaque volume (TPV) stages and the RF-CL subgroups.

**Fig. A3:**
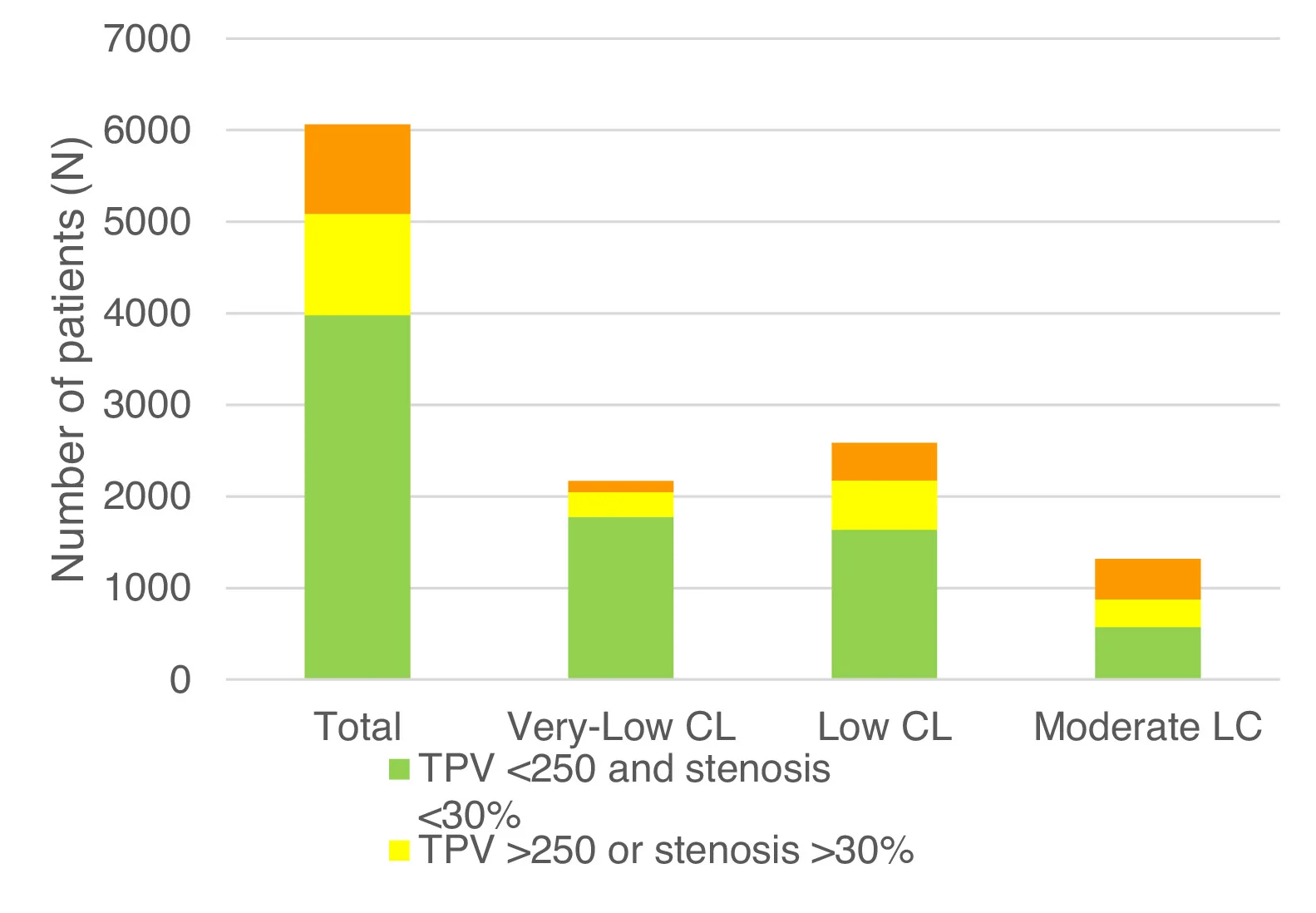
Distribution of patients in the total cohort and subdivided according to the three Risk Factor Weighted Clinical Likelihood groups.
