# Supplementary material for: Whole heart atherosclerosis volume and risk for major adverse cardiovascular events across the clinical likelihood for obstructive CAD; the CONFIRM2 study
Source: Am J Prev Cardiol. 2026 Jun 24;31:101712. doi: 10.1016/j.ajpc.2026.101712 (PMC13676140; doi:10.1016/j.ajpc.2026.101712)
Supplement: Supplementary file 1 [file mmc1.docx]

| **Appendix Table 1: Quantitative CT characteristics by RF-CL** | | | | | |
| --- | --- | --- | --- | --- | --- |
|  | **All Patients  N = 6061** | **Very Low  2161(36.0%)** | **Low  2585(43.0%)** | **Moderate  1315(22.0%)** | **P-value** |
| **Plaque volumes** |  |  |  |  |  |
| Total Plaque Volume, mm3 |  |  |  |  | <.0001* |
| Median (Q1, Q3) | 57.20 (16.20, 177.60) | 25.20 (7.30, 73.80) | 68.90 (21.90, 184.80) | 157.80 (55.70, 368.10) |  |
| Percent atheroma volume (PAV), % |  |  |  |  | <.0001* |
| Median (Q1, Q3) | 2.14 (0.63, 6.41) | 1.03 (0.32, 2.94) | 2.45 (0.82, 6.60) | 5.32 (1.80, 11.85) |  |
| Non-Calcified Plaque Volume, mm3 |  |  |  |  | <.0001* |
| Median (Q1, Q3) | 40.10 (12.20, 107.90) | 20.10 (5.70, 53.00) | 45.30 (15.60, 111.10) | 95.00 (34.90, 204.70) |  |
| Calcified Plaque Volume, mm3 |  |  |  |  | <.0001* |
| Median (Q1, Q3) | 8.10 (0.10, 55.90) | 1.00 (0.00, 12.00) | 12.40 (0.40, 61.80) | 42.00 (7.10, 156.00) |  |
| **Stenosis** |  |  |  |  |  |
| Quantitative Diameter stenosis, % |  |  |  |  | <.0001* |
| 0% | 580 (9.6%) | 362 (16.8%) | 182 (7.0%) | 36 (2.7%) |  |
| 1-24% | 3043 (50.2%) | 1281 (59.3%) | 1265 (48.9%) | 497 (37.8%) |  |
| 25-49% | 1514 (25.0%) | 371 (17.2%) | 732 (28.3%) | 411 (31.3%) |  |
| 50-69% | 526 (8.7%) | 88 (4.1%) | 244 (9.4%) | 194 (14.8%) |  |
| 70-99% | 272 (4.5%) | 43 (2.0%) | 116 (4.5%) | 113 (8.6%) |  |
| 100% | 126 (2.1%) | 16 (0.7%) | 46 (1.8%) | 64 (4.9%) |  |
| TPV staging system, mm3 |  |  |  |  | <.0001* |
| 0 | 435 (7.2%) | 270 (12.5%) | 136 (5.3%) | 29 (2.2%) |  |
| >0-250 | 4541 (74.9%) | 1749 (80.9%) | 1992 (77.1%) | 800 (60.8%) |  |
| >250-750 | 908 (15.0%) | 126 (5.8%) | 397 (15.4%) | 385 (29.3%) |  |
| >750 | 177 (2.9%) | 16 (0.7%) | 60 (2.3%) | 101 (7.7%) |  |
| NCPV Tertiles |  |  |  |  | <.0001* |
| 0-19.2 | 2020 (33.3%) | 1059 (49.0%) | 757 (29.3%) | 204 (15.5%) |  |
| >19.2-76.2 | 2022 (33.4%) | 729 (33.7%) | 929 (35.9%) | 364 (27.7%) |  |
| >76.2 | 2019 (33.3%) | 373 (17.3%) | 899 (34.8%) | 747 (56.8%) |  |
| NCPV |  |  |  |  | <.0001* |
| 0-20 | 2057 (33.9%) | 1075 (49.7%) | 772 (29.9%) | 210 (16.0%) |  |
| >20-125 | 2679 (44.2%) | 872 (40.4%) | 1230 (47.6%) | 577 (43.9%) |  |
| >125-375 | 1099 (18.1%) | 192 (8.9%) | 498 (19.3%) | 409 (31.1%) |  |
| >375 | 226 (3.7%) | 22 (1.0%) | 85 (3.3%) | 119 (9.0%) |  |
| Standard CAD classes |  |  |  |  | <.0001* |
| <50% stenosis | 5225 (86.2%) | 2034 (94.1%) | 2215 (85.7%) | 976 (74.2%) |  |
| 1-V >50% stenosis | 606 (10.0%) | 95 (4.4%) | 275 (10.6%) | 236 (17.9%) |  |
| 2-V >50% stenosis | 164 (2.7%) | 30 (1.4%) | 69 (2.7%) | 65 (4.9%) |  |
| 3-V >50% stenosis | 66 (1.1%) | 2 (0.1%) | 26 (1.0%) | 38 (2.9%) |  |
| Prox LAD or LM >50% stenosis | 461 (7.6%) | 67 (3.1%) | 199 (7.7%) | 195 (14.8%) | <.0001* |
| High Risk Plaque |  |  |  |  | <.0001* |
| 0 | 5725 (94.5%) | 2098 (97.1%) | 2440 (94.4%) | 1187 (90.3%) |  |
| 1 | 100 (1.6%) | 22 (1.0%) | 51 (2.0%) | 27 (2.1%) |  |
| >1 | 236 (3.9%) | 41 (1.9%) | 94 (3.6%) | 101 (7.7%) |  |

| **Appendix Table 2A: 4-Year Event Rates for the Primary Endpoint (MACE)** | | | | |
| --- | --- | --- | --- | --- |
|  | **Overall Patients   N = 6061** | **Very Low  2161(36.0%)** | **Low  2585(43.0%)** | **Moderate  1315(22.0%)** |
| Stenosis and TPV |  |  |  |  |
| <30% stenosis and TPV<250 | 90/3980 (2.3%) | 32/1766 (1.8%) | 39/1638 (2.4%) | 19/576 (3.3%) |
| >=30% stenosis or TPV>=250 | 71/1109 (6.4%) | 11/271 (4.1%) | 37/537 (6.9%) | 23/301 (7.6%) |
| >=30% stenosis and TPV>=250 | 142/972 (14.6%) | 16/124 (12.9%) | 59/410 (14.4%) | 67/438 (15.3%) |

| **Appendix Table 2B: 4-Year Event Rates for the Secondary Endpoint (Death/MI/Stroke)** | | | | |
| --- | --- | --- | --- | --- |
|  | **Overall Patients   N = 6061** | **Very Low  2161(36.0%)** | **Low  2585(43.0%)** | **Moderate  1315(22.0%)** |
| Stenosis and TPV |  |  |  |  |
| <30% stenosis and TPV<250 | 54/3980 (1.4%) | 25/1766 (1.4%) | 18/1638 (1.1%) | 11/576 (1.9%) |
| >=30% stenosis or TPV>=250 | 29/1109 (2.6%) | 6/271 (2.2%) | 13/537 (2.4%) | 10/301 (3.3%) |
| >=30% stenosis and TPV>=250 | 55/972 (5.7%) | 9/124 (7.3%) | 22/410 (5.4%) | 24/438 (5.5%) |
